# Psychometric Properties of the Digital Well-Being Scale and Its Links to Fear of Missing Out and Digital Identity

**DOI:** 10.3390/bs16010050

**Published:** 2025-12-26

**Authors:** Talía Gómez Yepes, Edgardo Etchezahar, Joaquín Ungaretti, María Laura Sánchez Pujalte

**Affiliations:** 1 Departamento de Psicología Evolutiva y de la Educación, Facultad de Formación del Profesorado y Educación, Universidad Autónoma de Madrid, Campus Cantoblanco, C/Francisco Tomás y Valiente, 3, 28049 Madrid, Spain; talia.gomezy@uam.es (T.G.Y.); joaquin.ungaretti@uam.es (J.U.); 2 Facultad de Educación, Universidad Internacional de Valencia, Calle del Pintor Sorolla, 21, Ciutat Vella, 46002 Valencia, Spain; marialaura.sanchez@professor.universidadviu.com

**Keywords:** digital well-being, fear of missing out, identity bubbles, validation, technology use

## Abstract

Digital well-being refers to the subjective balance between the benefits and drawbacks of technological connectivity. Although it is a relatively recent construct, research has shown that it can be measured reliably. The Digital Well-Being Scale, comprising three dimensions—Digital Satisfaction, Digital Wellness, and Safe and Responsible Behavior—has been validated in other countries, but not yet in Argentina. This study aimed to adapt and validate the scale in the Argentine context and to examine its associations with Fear of Missing Out (FoMO) and identity bubbles, two variables previously linked to digital experiences. A total of 895 participants (55.2% women; aged 18–65) completed an online survey including the Digital Well-Being Scale, the FoMO Scale, and the Identity Bubble Reinforcement Scale (IBRS-9). Exploratory and confirmatory factor analyses supported the original three-factor structure, and all dimensions showed an adequate internal consistency. A significant negative correlation was found between FoMO and the Digital Wellness dimension, suggesting that individuals with higher FoMO experience lower emotional balance in their digital lives. In contrast, associations between identity bubble dimensions and digital well-being were modest and selective. Only Digital Satisfaction and Digital Wellness were weakly related to social identification and homophily; no relationship was observed with safe digital behavior. These findings support the adapted scale’s psychometric soundness in the Argentine context and provide initial insights into how FoMO and digital identity processes may influence digital well-being. Further research is needed to explore these relationships in more diverse populations and cultural contexts.

## 1. Introduction

Over the last decades, digital technologies have become deeply embedded in everyday life, transforming how individuals work, learn, communicate, and engage in leisure activities (Ling, 2012; Roffarello & De Russis, 2019). Smartphones, computers, and networked platforms enable permanent access to information and social interaction, fostering a condition of ubiquitous connectivity in which being online is perceived as the norm and disconnection becomes salient only in its absence (Albalá-Genol et al., 2025; Vanden Abeele et al., 2018).

This structural transformation of daily life has prompted growing scholarly interest in understanding how individuals can maintain a healthy and sustainable relationship with technology. Rather than questioning the presence of digital media per se, contemporary research increasingly focuses on how connectivity can be integrated into everyday routines in ways that support psychological functioning and quality of life. Within this context, the concept of digital well-being has emerged as a central analytical framework.

### 1.1. Conceptualizing Digital Well-Being Beyond Problematic Technology Use

Early approaches to digital well-being tended to define the construct implicitly, often by contrast with maladaptive patterns of technology use such as internet or smartphone addiction (Durao et al., 2021; Etchezahar et al., 2023; Kardefelt-Winther, 2014; Pies, 2009). From this perspective, digital well-being was frequently equated with the absence of loss of control or functional impairment associated with excessive use. Although this view contributed to identifying risk behaviors, it offered a limited understanding of individuals’ lived digital experiences.

More recent theoretical developments emphasize that digital technologies can also provide meaningful benefits, including social connection, autonomy support, enjoyment, and functional assistance in daily life (Deci & Ryan, 2008; Henderson & Knight, 2012; Huta, 2016). In line with broader theories of subjective well-being, which conceptualize well-being as dynamic, experiential, and context-dependent (Diener et al., 1999; Headey & Wearing, 1989), digital well-being is increasingly understood as a subjective evaluation of how digital connectivity contributes to—or detracts from—one’s overall quality of life.

Vanden Abeele (2021) proposed an integrative and influential definition of digital well-being as the individual and subjective experience of achieving an optimal balance between the benefits and the drawbacks of digital connectivity. This balance involves both affective and cognitive appraisals of technology use and acknowledges that positive and negative digital experiences may coexist. Under this framework, digital well-being is not a static state but a dynamic process shaped by personal characteristics, social contexts, and situational demands.

### 1.2. Measurement Approaches to Digital Well-Being

Alongside conceptual advances, several instruments have been developed to operationalize digital well-being and related constructs. Early measurement efforts often focused on specific skills or risk perceptions within digital environments. For example, Öztürk (2018) introduced a Digital Well-Being Scale emphasizing individuals’ ability to manage digital platforms and share personal information responsibly, reporting satisfactory psychometric properties and a positive association with subjective well-being. Similarly, Arslankara and Usta (2018) developed the Virtual World Risk Perception Scale to assess perceived threats and opportunities in online contexts, while Korkmaz et al. (2021) proposed the Online Privacy Awareness Scale, targeting attention, security, and communication dimensions.

Other instruments conceptualized digital well-being through patterns of media use. Kara (2019), for instance, assessed digital well-being among university students by focusing on different types of social media usage, whereas Mathew et al. (2023) developed a multidimensional scale capturing physical, mental, and emotional aspects of well-being in young adults. Although these tools demonstrated adequate psychometric quality, they often operationalized digital well-being indirectly or within restricted populations, limiting their capacity to capture the subjective trade-offs inherent in continuous connectivity.

Addressing these limitations, Arslankara et al. (2022) developed a comprehensive Digital Well-Being Scale grounded in a multidimensional and experiential understanding of the construct. The scale comprises three interrelated dimensions: Digital Satisfaction, reflecting enjoyment and positive engagement with digital technologies; Digital Wellness, capturing emotional and cognitive balance in digital interactions; and Safe and Responsible Behavior, assessing protective and self-regulatory practices online. This instrument showed satisfactory validity and reliability and offers a theoretically coherent framework aligned with contemporary conceptualizations of digital well-being.

### 1.3. Digital Well-Being in Relation to Fear of Missing Out and Digital Identity Processes

Beyond measurement, recent research has examined digital well-being in relation to psychological processes that shape online engagement. One of the most prominent constructs in this regard is Fear of Missing Out (FoMO), defined as a pervasive apprehension that others might be having rewarding experiences from which one is absent (Przybylski et al., 2013). FoMO has been consistently linked to increased social media use, anxiety, sleep disturbances, and lower life satisfaction (Andreassen, 2015; Tandon et al., 2021; Ungaretti et al., 2025). While FoMO may motivate social connection, empirical evidence suggests that it often undermines emotional balance and subjective well-being, making it a relevant correlate of digital well-being.

Another relevant line of research concerns digital identity and the formation of so-called identity bubbles. According to Keipi et al. (2017) and Kaakinen et al. (2018), identity bubbles emerge when social media users interact primarily with like-minded others, reinforced by algorithmic content personalization. These environments are characterized by strong social identification, homophily, and information bias, which can simultaneously provide social validation and restrict exposure to diverse perspectives. Previous studies indicate that such dynamics may influence attitudes, beliefs, and well-being in complex and context-dependent ways (Gómez Yepes et al., 2024; Savolainen et al., 2020).

### 1.4. The Present Study

Despite growing interest in digital well-being, validated instruments remain scarce in Latin American contexts. Moreover, there is limited empirical evidence on how digital well-being relates to FoMO and identity bubble processes within the general adult population. To address these gaps, the present study aims to adapt and validate the Digital Well-Being Scale developed by Arslankara et al. (2022) for use in Argentina. In addition, to provide initial evidence of criterion-related validity, this study examines the associations between digital well-being, Fear of Missing Out, and digital identity bubbles. By integrating these lines of research, the study seeks to contribute to a more coherent and theoretically grounded understanding of digital well-being in contemporary, highly connected societies.

## 2. Materials and Methods

### 2.1. Measures

#### 2.1.1. Participants

A nationwide online questionnaire was administered employing stratified sampling based on the geographical regions of Argentina. A total of 895 complete and valid responses were collected, with 55.2% (*n* = 494) participants identifying as female and 44.8% (*n* = 401) as male. Participants’ ages ranged from 18 to 65 years, with a mean age of 48.40 years (*SD* = 11.80). Regarding educational attainment, 9.4% reported primary education, 39.7% secondary education, 28.8% higher non-university tertiary education, and 22.1% university-level education. In terms of self-reported social class, 10.9% identified as lower class, 31.8% as lower-middle class, 49.6% as middle class, and 7.7% as upper-middle class.

#### 2.1.2. Measures

***Digital Well-Being.*** We used the scale developed by Arslankara et al. (2022), which consists of 12 items grouped into three dimensions: Digital Satisfaction (e.g., “I am interested in new digital experiences that can provide different sensations,” “I enjoy spending time with digital technologies”), Digital Wellness (e.g., “I relate better to people through social networks,” “I feel bad when my posts do not reach a certain number of likes or interactions”—reverse coded), and Safe and Responsible Behavior (e.g., “I avoid sharing sensitive personal information on digital platforms,” “I always act with caution regarding any harm that may occur to me in the digital world”). The items were rated on a 5-point Likert scale ranging from 1 = Totally disagree to 5 = Totally agree. Previous studies have shown satisfactory psychometric properties for this scale.

***Fear of Missing Out (FoMO).*** The construct was assessed using an adapted version of the original FoMO scale developed by Przybylski et al. (2013). The scale consists of 10 items designed to measure individuals’ pervasive apprehension that others might be having rewarding experiences from which they are absent. Although the original validation proposed a two-factor structure, consistent with recent literature suggesting a unidimensional interpretation for broader applications (e.g., Muller et al., 2024; Durao et al., 2023). Participants responded to each item using a 5-point Likert scale ranging from 1 (Strongly disagree) to 5 (Strongly agree). Sample items include “I fear others have more rewarding experiences than me,” “It is important that I understand what my friends are doing,” and “When I miss out on a planned get-together, it bothers me.” Higher scores indicate a higher level of FoMO. In our sample, the scale’s reliability was adequate (α = 0.82).

***Identity Bubbles.*** We used the 9-item Identity Bubble Reinforcement Scale (IBRS-9; Kaakinen et al., 2018) to assess participation in identity bubbles on social networks. Responses are given on a five-point scale (1 = Totally disagree, 5 = Totally agree). The IBRS-9 has three correlated factors: Social Identification (e.g., “I feel a strong sense of belonging with my social media contacts”), Homophily (e.g., “I prefer to follow people who share my values”), and Information Bias (e.g., “The information I receive on social media reinforces my views”). The original study (Kaakinen et al., 2018) provided evidence for this three-factor structure (RMSEA = 0.047, 90% CI [0.029, 0.065]; CFI = 0.988; TLI = 0.982) and reported adequate internal consistency for each factor (0.79 < α < 0.92).

***Adaptation and Interest in New Technologies*.** We included two single-item measures: “Adaptation to new technologies” and one for “Interest in new digital experiences” (Meerkerk et al., 2009). Both items were rated on a five-point Likert scale (1 = totally disagree, 5 = totally agree).

***Socio-demographic data questionnaire*.** Participants reported their gender, age, self-perceived socio-economic level, and highest educational level.

#### 2.1.3. Procedure and Data Analysis

Participants who met the inclusion criteria (aged 18 years or older and residing in specified geographic regions) were invited to participate via social media using a quota sampling strategy. Prior to participation, individuals were informed about the objectives of the study, the institution responsible for its implementation, and were provided with an email address for inquiries. They were also assured that all data would be used exclusively for academic and scientific purposes and handled in accordance with Argentina’s National Personal Data Protection Law (Law No. 25,326). The scales, originally in English, were translated into Spanish by a team of bilingual researchers using a back-translation procedure to ensure conceptual equivalence. This process was carried out in March 2024.

Statistical analyses were conducted using SPSS for Windows, version 19.0 (George & Mallery, 2010), and EQS 6.1 (Bentler, 2007). The sample size (*n* = 895) is large, which provides high statistical power. However, it is acknowledged that for factor analysis, such a large sample might lead to an over-rejection of models that have an acceptable fit in smaller samples. We proceeded with the analysis, keeping in mind that even minor discrepancies could become statistically significant. A confirmatory factor analysis (CFA) was performed to assess the factorial structure of the scale. Descriptive statistics (mean, standard deviation, skewness, and kurtosis) were calculated for each item in the final version of the scale. Internal consistency was examined using Cronbach’s alpha. Construct validity was assessed through an exploratory factor analysis with varimax rotation, followed by a comparison of two SEM models: a one-factor model and a correlated three-factor model of the construct. Lastly, to evaluate criterion validity, correlations between the dimensions of Digital Well-Being and related constructs—including Fear of Missing Out (FoMO), identity bubbles, and adaptation to and interest in new technologies—were analyzed.

## 3. Results

First, the descriptive statistics and reliability of the Digital Well-Being Scale and its three dimensions were analyzed (Table 1).

As shown in Table 1, skewness and kurtosis values for all items fell within acceptable ranges. Regarding reliability, the deletion of any item did not improve the Cronbach’s alpha for its respective dimension, and all item-total correlations were satisfactory (*r* > 0.35) (Hair et al., 2016). Furthermore, exploratory factor analysis (KMO = 0.736; Bartlett’s test, *p* < 0.001) supported a three-factor structure. The total variance explained was 58.55%, with the first factor accounting for 21.51%, the second for 18.65%, and the third for 18.39% of the variance.

Concerning construct validity, the original model proposed by Arslankara et al. (2022) consisted of three dimensions. However, it was necessary to examine whether a unidimensional model encompassing all items could adequately fit the data. To address this, two confirmatory factor analyses (CFAs) were conducted to compare the fit of the one-factor and three-factor models (see Table 2).

**Figure 1:**
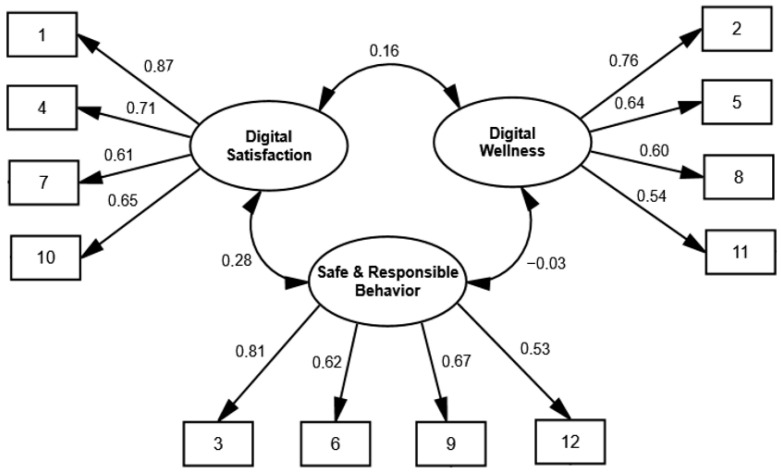
This Confirmatory Factor Analysis of the three-dimensional model of the Digital Well-Being Scale.

Table 2 indicates that all fit indices were satisfactory for the three-dimensional correlated model, whereas the unidimensional model of the Digital Well-Being Scale demonstrated poor fit to the data. Figure 1 presents the factor loadings of each item on its respective dimension within the three-factor model.

Next, following the analysis of descriptive statistics and the assessment of the scale’s reliability and validity, the relationships between the three Digital Well-Being dimensions and the variables of gender and age were examined. The results revealed a significant gender difference in the Safe and Responsible Behavior dimension (*t* = 2.354; *p* < 0.05; Cohen’s *d* = 0.263), with females (*M* = 4.23, *SD* = 0.85) scoring higher than males (*M* = 4.01, *SD* = 0.82). No significant gender differences were found in the other two dimensions, nor were there any significant differences across age groups for any of the three dimensions.

Subsequently, the associations between the three Digital Well-Being dimensions and related constructs as the Fear of Missing Out (FoMO), the dimensions of digital identity bubbles (Homophily, Social Identification, and Information Bias), as well as levels of adaptation to new technologies and interest in novel digital experiences—were analyzed (see Table 3).

As presented in Table 3, the dimensions of Digital Well-Being demonstrated significant associations with several external variables. Fear of Missing Out (FoMO) exhibited a strong negative relationship with the “Digital Wellness” dimension and a moderate association with “Digital Satisfaction.” Furthermore, these two dimensions of Digital Well-Being were significantly correlated with key aspects of digital identity, including Homophily, Social Identification, and Information Bias. Lastly, both adaptation to new technologies and interest in novel digital experiences were positively linked to the “Safe and Responsible Behavior” dimension as well as to “Digital Satisfaction,” suggesting these factors play a meaningful role in shaping individuals’ responsible engagement and overall satisfaction in digital environments.

The non-significant correlation between Digital Wellness and Safe and Responsible Behavior is an interesting finding. It may suggest that feeling emotionally balanced in the digital world does not necessarily translate into safer online practices, indicating that these are distinct psychological domains that may require different intervention strategies.

## 4. Discussion

This study aimed to validate an adapted version of the Digital Well-Being Scale within the Argentine context, and the results confirm that the instrument exhibits sound psychometric properties. The findings provide robust evidence of construct validity and internal consistency, affirming that the original 12-item, three-dimensional structure, comprising Digital Satisfaction, Digital Wellness, and Safe and Responsible Behavior, is a suitable and reliable framework for assessing digital well-being in this new cultural setting. The successful adaptation of this scale represents a significant contribution to the field, as it addresses a notable gap in the literature concerning the psychometric measurement of digital well-being in Latin American populations. By providing a validated tool, this research opens the door for more nuanced investigations into the complex interplay between technology use and psychological health in a region with unique patterns of digital adoption and engagement.

### 4.1. Psychometric Properties of the Digital Well-Being Scale

The confirmation of the three-factor structure is a pivotal finding, as it aligns with the original research by Arslankara et al. (2022) and suggests a degree of universality in the conceptualization of digital well-being. The stability of these dimensions across different cultural contexts implies that the core components of digital well-being, subjective satisfaction with digital life, positive emotional and cognitive states online, and protective behaviors, are fundamental aspects of this construct. This consistency is particularly noteworthy given the challenges inherent in the cross-cultural adaptation of psychological instruments, where linguistic and cultural nuances can often alter the underlying factor structure. The successful replication in an Argentine sample reinforces the conceptual validity of digital well-being as a multidimensional construct and provides a solid foundation for comparative research across diverse populations.

A particularly telling finding emerged from the analysis of gender differences. While no significant variations were observed in the Digital Satisfaction and Digital Wellness dimensions, women scored significantly higher than men on Safe and Responsible Behavior. This finding is more than a simple replication of previous literature on gender-based differences in online attitudes; it points toward a deeper divergence in how different genders navigate the digital world. This pattern may be rooted in differential socialization processes, where women are often encouraged to be more cautious and risk-averse (Savolainen et al., 2020). Furthermore, it may reflect a pragmatic response to the documented higher prevalence of online risks faced by women, such as cyberstalking and harassment, which necessitates the adoption of more stringent safety measures (Kaakinen et al., 2020). The absence of gender differences in the emotional and satisfaction-based dimensions of digital well-being is equally significant. It suggests that while behavioral strategies may differ, the fundamental emotional experience of digital life may be more homogenous across genders. This highlights the necessity for a nuanced approach to digital literacy and safety interventions, one that acknowledges shared emotional experiences while addressing gender-specific behavioral patterns and risks.

In examining criterion validity, this study uncovered a significant negative correlation between Fear of Missing Out (FoMO) and the emotional–cognitive dimensions of digital well-being (Digital Wellness and, to a lesser extent, Digital Satisfaction). This aligns with a growing body of research that frames FoMO as a source of digital-related anxiety and diminished life satisfaction (Durao et al., 2023). Our findings deepen this understanding by revealing a critical dissociation: FoMO was not significantly related to the behavioral dimension of Safe and Responsible Behavior. This suggests a complex psychological mechanism at play. FoMO appears to erode the emotional quality of digital experiences, likely by fostering a state of hyper-vigilance and social anxiety, yet it does not necessarily translate into a disregard for online safety (Tandon et al., 2021). One possible explanation is that FoMO drives an increase in the quantity of online engagement, which in turn amplifies exposure to emotionally taxing content and social comparisons, thereby diminishing well-being (Andreassen, 2015). However, the quality of that engagement, in terms of safety practices, may remain intact, governed by established knowledge and routines rather than fluctuating emotional states. This points to the potential role of self-regulation as a mediating factor; while individuals may struggle to regulate the emotional impact of constant connectivity driven by FoMO, their learned safety protocols remain robust (Gómez Yepes et al., 2025; Muller et al., 2024).

Perhaps the most complex set of findings relates to the role of digital identity bubbles. The study found only small, albeit significant, correlations between the identity bubble dimensions (Homophily, Social Identification, and Information Bias) and the emotional facets of digital well-being, with no relationship to Safe and Responsible Behavior. This presents a fascinating contrast to recent research, such as that by Fan et al. (2024), which found a positive association between identity bubble reinforcement and subjective happiness among Chinese medical professionals. This discrepancy is not a contradiction but rather a crucial point of dialogue that underscores the context-dependent nature of digital identity’s impact on well-being (Savolainen et al., 2020).

The paradox of the identity bubble, its ability to provide social support while simultaneously fostering cognitive biases and limiting exposure to diverse perspectives, may resolve differently depending on the population in question (Tandon et al., 2021; Ungaretti et al., 2025). For the general Argentine population in our sample, the modest and somewhat ambivalent relationship with well-being may suggest that the benefits of social validation within these bubbles are counteracted by the negative effects of informational echo chambers and potential polarization. In contrast, for a specialized, high-stress professional group like the medical practitioners in the study by Fan et al. (2024), the social support and shared identity aspects of a digital bubble might offer a crucial buffer against occupational stress, making the overall effect on well-being more positive. This highlights that the architecture of our digital social lives is not inherently good or bad; its impact on our well-being is mediated by our social context, our profession, and our cultural environment. Future research must move beyond asking if identity bubbles are harmful and instead explore for whom and under what circumstances they support or undermine well-being.

### 4.2. Theoretical Implications for the Conceptualization of Digital Well-Being

The collective findings of this study have several important theoretical and practical implications. Theoretically, our results reinforce the conceptualization of digital well-being as a multifaceted construct. The clear divergence in how the emotional–cognitive dimensions (Wellness and Satisfaction) and the behavioral dimension (Safe and Responsible Behavior) relate to external variables like FoMO and identity bubbles provides strong evidence against a monolithic view of digital well-being. It suggests that different psychological processes underpin these distinct facets of our digital lives. This nuanced model is critical for advancing theory in the field, moving from broad-stroke associations to more precise models of how specific digital experiences and predispositions interact to shape an individual’s overall sense of well-being in a connected world.

Practically, these findings offer a roadmap for designing more targeted and effective interventions. The strong link between FoMO and diminished emotional well-being suggests that interventions should focus on building emotional regulation skills and promoting mindful technology use. Techniques drawn from cognitive-behavioral therapy (CBT) or mindfulness-based stress reduction (MBSR) could be adapted to help individuals manage the anxiety and social pressure associated with FoMO. The gender-specific findings in safety behaviors indicate that a one-size-fits-all approach to digital literacy is insufficient. While all users need foundational safety knowledge, interventions may need to be tailored to address the unique risks and social pressures faced by women. Finally, the complex role of identity bubbles suggests that interventions should focus on fostering critical media literacy. Rather than simply encouraging users to ‘break their bubbles,’ a more effective approach may be to equip them with the skills to critically evaluate information, recognize algorithmic bias, and engage with diverse perspectives in a constructive manner.

### 4.3. Limitations and Future Research Directions

Despite its contributions, this study is not without limitations. First, the reliance on self-report measures is a notable constraint. While standard in psychological research, these measures are susceptible to social desirability bias and inaccuracies in self-perception and memory. Future research would be greatly enhanced by incorporating objective, behavioral data. For instance, with participant consent, screen time data from smartphones or browser extensions could provide a more accurate measure of digital engagement, which could then be correlated with self-reported well-being. Second, the cross-sectional design of the study precludes any conclusions about causality. It is impossible to determine, for example, whether high FoMO leads to lower digital well-being or if individuals with pre-existing low well-being are more susceptible to FoMO. Longitudinal studies that track individuals over time are essential to unraveling the directional nature of these relationships. A prospective study following a cohort of young adults through a significant life transition, such as entering university, could provide invaluable insights into the dynamic interplay between digital habits and well-being.

Furthermore, the sampling methodology, while efficient, presents limitations. The use of online recruitment via social media may have resulted in a sample that is not fully representative of the broader Argentine population, potentially over-representing individuals who are more digitally savvy or have higher levels of education. The relatively high average age of our sample also means that the experiences of younger digital natives—a crucial demographic in any discussion of digital life—may be underrepresented. Future research should strive for more diverse and representative sampling methods, including community-based recruitment, to capture a fuller spectrum of the population. Finally, future studies should expand the scope of inquiry to include qualitative methods. While quantitative data provides an excellent overview of the ‘what,’ qualitative approaches such as in-depth interviews or digital ethnography are needed to understand the ‘why.’ Listening to individuals’ lived experiences of navigating FoMO, managing their digital identities, and striving for well-being would provide a rich, contextualized understanding that can complement and bring to life the statistical patterns observed in this study.

## Figures and Tables

**Table 1:** Descriptive statistics, reliability, and exploratory factor analysis of the Digital Well-Being Scale.

Digital Satisfaction (α = 0.801)	*M*	*SD*	*S*	*K*	ri.t	α − i	1	2	3
1. Me interesan las nuevas experiencias digitales que pueden brindar diferentes vivencias (*I am interested in new digital experiences that can offer different kinds of experiences*)	3.50	1.23	−0.73	−0.31	0.717	0.698	**0.853**	0.100	0.115
4. Disfruto pasar tiempo con las tecnologías digitales (*I enjoy spending time with digital technologies*).	3.69	1.13	−0.87	0.12	0.594	0.761	**0.773**	0.143	0.078
7. Puedo adaptarme fácilmente a las nuevas tecnologías (*I can easily adapt to new technologies*).	3.61	1.24	−0.75	−0.46	0.575	0.770	**0.772**	−0.143	0.076
10. En habilidades digitales, me siento en armonía con las personas que me rodean (*In digital skills, I feel in harmony with the people around me*).	3.17	1.21	−0.31	−0.74	0.575	0.770	**0.736**	0.121	0.174
**Digital Wellness (α = 0.729)**	*M*	*SD*	*S*	*K*	ri.t	α − i	1	2	3
2. Me preocupa recibir comentarios negativos en redes sociales (*I worry about receiving negative comments on social media*).	2.65	1.31	0.06	−1.17	0.606	0.616	0.067	**0.800**	0.073
5. Me hace sentir mal cuando mis publicaciones no alcanzan determinado número de likes o interacciones (*It makes me feel bad when my posts don’t reach a certain number of likes or interactions*).	1.89	1.17	0.94	−0.30	0.531	0.665	−0.026	**0.741**	−0.069
8. Si me expreso libremente en las redes sociales, pienso que seré excluido(a) por algunas personas en mis redes sociales (*If I express myself freely on social media, I think I will be excluded by some people in my social networks*).	2.77	1.40	0.01	−1.29	0.489	0.690	−0.008	**0.717**	−0.060
11. Me relaciono mejor con las personas a través de las redes sociales (*I relate better to people through social media*).	2.20	1.28	0.56	−0.93	0.463	0.701	0.179	**0.658**	0.047
**Safe and Responsible Behavior (α = 0.730)**	*M*	*SD*	*S*	*K*	ri.t	α − i	1	2	3
3. Cuido mi privacidad y configuro adecuadamente las opciones de privacidad en mis perfiles y cuentas en línea (*I take care of my privacy and properly configure the privacy settings on my online profiles and accounts*),	4.01	1.03	−1.68	1.39	0.637	0.605	0.089	−0.067	**0.828**
6. Respaldo periódicamente mis datos y archivos digitales para evitar pérdidas irreparables (*I regularly back up my data and digital files to avoid irreparable loss*).	3.71	1.28	−0.74	−0.48	0.522	0.676	0.114	0.076	**0.722**
9. Evito compartir información personal sensible en plataformas digitales (*I avoid sharing sensitive personal information on digital platforms*).	4.14	0.96	−1.43	1.43	0.520	0.675	−0.002	−0.130	**0.721**
12. Siempre actúo con precaución ante cualquier daño que pueda ocurrirme en el mundo digital (*I always act cautiously to prevent any harm that could happen to me in the digital world*).	4.13	1.11	−1.33	1.14	0.436	0.718	0.245	0.107	**0.639**

***Note.*** *M*: Mean; *SD*: Standard Deviation; *S*: Skewness; *K*: Kurtosis; r.it: item–total correlation; α − i: alpha if item deleted. Items in English in parentheses.

**Table 2:** Confirmatory Factor Analysis fit indices for one-dimensional vs. three-dimensional models of the Digital Well-Being Scale.

	S-B χ^2^ (gl)	ΔS-B χ^2^ (gl)	CFI	IFI	RMSEA
One-dimensional model of the Digital Well-being Scale	799.28 (54)	14.80	0.470	0.479	0.124 [0.117–0.132]
Three-dimensional correlated model of the Digital Well-being Scale	159.956 (51)	3.13	0.923	0.924	0.049 [0.040–0.058]

***Note.*** Acceptable values: ΔS-B χ^2^ (gl) ≤ 5; CFI, IFI ≥ 0.90; RMSEA ≤ 0.08.

**Table 3:** Pearson correlations between Digital Well-Being dimensions and related variables.

	1	2	3	4	5	6	7	8	9
1. Digital Satisfaction	0.801	0.140 **	0.268 **	−0.177 **	0.076	0.214 **	0.245 **	0.272 **	0.255 **
2. Digital Wellness		0.729	0.016	−0.417 **	0.177 **	0.202 **	0.234 **	−0.009	0.137 **
3. Safe and Responsible Behavior			0.730	0.075	0.047	0.077	0.057	0.231 **	0.253 **
4. Fear of Missing Out				0.812	0.227 **	0.227 **	0.180 **	0.020	0.194 **
5. Homophily					0.809	0.479 **	0.383 **	−0.019	0.042
6. Social Identification						0.761	0.478 **	0.080	0.152 **
7. Information Bias							0.778	0.072	0.238 **
8. Adaptation to new technologies								0.805	0.510 **
9. Interest in new digital experiences									0.759

***Note.*** Cronbach’s alpha on the diagonal. ** *p* < 0.001.

## Data Availability

The raw data supporting the conclusions of this article will be made available by the authors upon request.
